# Social inequalities and cancer: can the European deprivation index predict patients' difficulties in health care access? a pilot study

**DOI:** 10.18632/oncotarget.6274

**Published:** 2015-11-02

**Authors:** Guillaume Moriceau, Aurélie Bourmaud, Fabien Tinquaut, Mathieu Oriol, Jean-Philippe Jacquin, Pierre Fournel, Nicolas Magné, Franck Chauvin

**Affiliations:** ^1^ Medical Oncology Lucien Neuwirth Cancer Institute, Saint Priest en Jarez, France; ^2^ Department of Public Health, Hygée Centre, Lucien Neuwirth Cancer Institute, Saint Priest en Jarez, France; ^3^ Therapeutic Targeting in Oncology, EMR3738, Claude Bernard University, Lyon, France; ^4^ Radiation Oncology Lucien Neuwirth Cancer Institute, Saint Priest en Jarez, France; ^5^ Clinical Investigation Center and Clinical Epidemiology, Jean Monnet University, Saint-Etienne, France

**Keywords:** cancer, access to care, socio-economic inequalities, european deprivation Index, population based

## Abstract

**Context:**

The European Deprivation Index (EDI), is a new ecological estimate for Socio-Economic Status (SES). This study postulates that Time-To-Treatment could be used as a cancer quality-of -care surrogate in order to identify the association between cancer patient's SES and quality of care in a French comprehensive cancer center.

**Methods:**

retrospective mono-centered cohort study. All consecutive incoming adult patients diagnosed for breast cancer(BC), prostate cancer(PC), colorectal cancer (CRC), lung cancer(LC) or sarcoma(S) were included between January 2013 and December 2013. The association of EDI and Time-To-Diagnosis(TTD), as well as Time-To-Treatment(TTT) was analyzed using a cox regression, and a strata analysis per tumor site was performed.

**Results:**

969 patients were included. Primitive tumor site was 505 BC(52%), 169 PC(17%), 145 LC(15%), 116 CRC(12%), and 34 S(4%). Median TTD was 1.41 months (Q1-Q3 0.5 to 3.5 months). Median TTT was 0.9 months (0.4 - 1.4). In a multivariate analysis, we identified the tumor site as a predictive factor to influence TTD, shorter for BC (0.75months, [0.30- 1.9]) than PC (4.69 months [1.6-29.7]), HR 0.27 95%CI= [0.22-0.34], *p* < 0.001. TTT was also shorter for BC (0.75months [0.4-1.1]) than PC (2.02 [0.9-3.2]), HR 0.32 95%CI= [0.27-0.39], *p* < 0.001. EDI quintiles were not found associated with either TTT or TTD.

**Conclusions:**

Deprivation estimated by the EDI does not appear to be related to an extension of the Time-to-Diagnosis or Time-to-Treatment in our real-life population. Further research should be done to identify other frailty-sensitive factors that could be responsible for delays in care.

## INTRODUCTION

In a redistributive health care system, the aim of social health insurance is to provide equal access to health care to all insurees, in order to reduce or at least to avoid creating more social inequalities. Yet this objective is not reached in numerous developed countries, benefiting from National Health Insurance. Evidence has been given to inequalities in health care and documented worldwide, especially in France, where social inequities are among the highest in Europe [1, 2].

Nowadays, cancer is the second cause of death by non-communicable diseases in the world and the first cause of mortality in France [3]. An association has been identified between patients ‘Socioeconomic Status’ (SES) and different cancer outcomes. Studies from various countries highlighted a significant impact of the SES on cancer diagnosis [4–6], treatment [7, 8], mortality [9–13] and cancer survivors' rehabilitation [14] : the lower the SES is, the worse the outcome is. Socioeconomic Status has also been demonstrated to be linearly related to cancer screening attendance [15–20]. But few studies observed this phenomenon in France [21–23]. Furthermore, few studies have evaluated the association between SES and the time between symptoms to diagnosis or between diagnosis to first hospital care [24, 25] and none of them are French. Yet these indicators have demonstrated to be good surrogates of quality-of-care [26–28].

The majority of the studies dealing with cancer inequities used an ecological index of social deprivation to estimate patients' socio-economic status in order to identify the relation between SES and cancer outcomes. The most used is the Townsend index. This index is defined as “a state of observable and demonstrable disadvantages relative to the local community or more widely to the society to whom the patient, family or group belongs” [29]. In France, the use of this Index to estimate SES highlighted a low impact of the “deprivation” on cancer outcomes [30]. The limits raised by this study were that this index was too rough to capture individual and community specificities.

Recently, a new ecological deprivation index, the European Deprivation Index (EDI), has been developed [31]. This Index is a better match to patients' cultural and social environment since it is constructed and tailored for each European country. But the EDI can also be replicated in 24 other European countries, and used to perform international comparisons.

We postulate that EDI could be a better estimator of SES than the Towsend Index. We also postulate that since time-to-treatment has demonstrated to be associated with quality of care, it could be used as a cancer outcome surrogate in this study. Thus, the objective of this study is to identify the association between cancer patient's deprivation and quality of care in a French comprehensive care centre. The principal objective was to measure the influence of patients' SES, estimated by the EDI, on the time-to-diagnosis (time between onset of symptoms and first histological evidence) in newly diagnosed patients. The secondary objectives were to identify associations between deprivation and Time-to-Treatment (time between diagnosis and first specialized medical or surgical treatment) and other outcomes.

## RESULTS

In civil year 2013, 969 patients were admitted to our institution for a diagnosis of BC, PC, CRC, LC or S. All of them had histological proved malignancies. Median age was 65 years (Table 1). 505 patients had breast cancer (52%), 169 had prostate cancer (17%), 145 had lung cancer (15%), 116 had colorectal cancer C (12%), and 34 had sarcoma (4%). 82% patients had no metastatic disease at diagnosis. Performance status at baseline was 0 for 612 patients (63%) and ≥ 3 for 21 patients (2%).

**Table 1:** Characteristics of patients

n		969
**Age (years)**		
	Median [Q1-Q3]	65 [55-74]
	Mean (SD)	63.9 (13.2)
	Range	19 to 92
**Gender**		
	Female	608 (63%)
	Male	361 (37%)
**Year of diagnosis**		
	2013	969 (100%)
**Primitive tumor**		
	Breast	505 (52%)
	Prostate	169 (17%)
	Lung	145 (15%)
	Colon or rectum	116 (12%)
	Sarcoma	34 (4%)
**T staging at diagnosis**		
	in situ	10 (1%)
	1	381 (39%)
	2	252 (26%)
	3	147 (15%)
	4	68 (7%)
	X	21 (2%)
	missing	75 (8%)
**N staging at diagnosis**		
	0	527 (54%)
	1	207 (21%)
	2	100 (10%)
	3	27 (3%)
	X	28 (3%)
	missing	67 (7%)
**M staging at diagnosis**		
	0	794 (82%)
	1	170 (18%)
	X	4 (0%)
	missing	1 (0%)
**Performance status at baseline**		
	0	612 (63%)
	1	257 (27%)
	2	72 (7%)
	≥3	21 (2%)
	missing	7 (1%)
**Deadline of referral, months**		
	Time to diagnosis, median [Q1-Q3]	1.41 [0.5-3.5]
	Time to treatment, median [Q1-Q3]	0.9 [0.4-1.4]
**First treatment started**		
	surgery	584 (60%)
	chemotherapy	148 (15%)
	hormone therapy	112 (12%)
	radiotherapy	65 (7%)
	radio-chemotherapy	40 (4%)
	best supportive care	8 (1%)
	missing	8 (1%)
**Last know status (at 1 January 2014)**		
	Death	33 (3%)
	Censored	350 (36%)
	Alive	586 (60%)
Ecological Deprivation Index*		
	Quintile 1: the least deprived	129 (13%)
	Quintile 2	201 (21%)
	Quintile 3	166 (17%)
	Quintile 4	185 (19%)
	Quintile 5: the most deprived	279 (29%)
	missing	9 (1%)

* Aggregate variable of the neighborhood, French version

**Figure 1:**
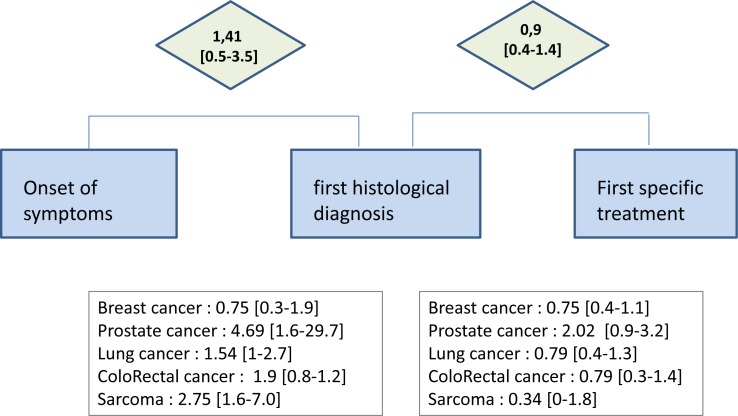
Times between care intervals for the all 969 cancer patients included in the study, newly diagnosed and treated in the Saint Etienne Comprehensive Cancer Center, between the first of January 2013 and the 31 of december 2013 Times-to-diagnosis and Time-to-treatement are also given by tumor site. Times are in months, median [interquartile].

Median Time-To-Diagnosis was 1.41 months [Q1-Q3 ranging 0.5 to 3.5 months] for all cancers (0.75 months [0.3-1.9] for BC, 4.69 months [1.6-29.7] for PC, 1.54 months [1-2.7] for LC, 1.90 months [0.8-1.2] for CRC and 2.75 months [1.6-7.0] for S (Figure 1). Median Time-To-Treatment was 0.9 month [Q1-Q3 ranging 0.4 to 1.4 months] for all cancers and 0.75 months [0.4-1.1] for BC, 2.02 months [0.9-3.2] for PC, 0.79 months [0.4-1.3] for LC, 0.79 months [0.3-1.4] for CRC and 0.34 months [0-1.8] for S (Figure 1). Surgery was the most frequent first treatment (*N* = 588, 61%), then chemotherapy (*N* = 148, 15%), hormonal therapy (*N* = 112, 12%) and radiotherapy (*N* = 65, 7%). 129 patients (13%) were included in the quintile 1 of the EDI (the least deprived), 201 (21%) in the quintile 2, 166 (17%) in the quintile 3, 185 (19%) in the quintile 4 and 279 patients (29%) lived in quintile 5- area, the most deprived.

Univariate associations between patients' characteristics and Time-To-Diagnosis are reported on Table 2. EDI quintile 2/3 are associated with a shorter Time-To-Diagnosis compared to quintile 5 (36 *vs* 48 days, HR 0.79, 95%CI = [0.65-0.96], *p* = 0.02). After elimination of correlated variables, multivariate analysis (Table 2) did not identify EDI as being associated with Time-To-Diagnosis (HR 0.96, 95%CI = [0.78-1.19], *p* = 0.426). Only the tumor site statistically influenced the Time-To-Diagnosis: The Time-to-Diagnosis is shorter for breast cancer (0.75months [0.30-1.9]) and longer for prostate cancer (4.69 months [1.6-29.7]) with an adjusted HR = 0.27(95%CI = [0.22-0.34], *p* < 0.001).

**Table 2:** Time-To-Diagnosis univariate and multivariate analyses

	Univariate analysis		Multivariate analysis	
	HR [95%CI]	*p value*	AHR [95%CI]	*p* value
**AGE**				
[19-55]	1 (reference)	*<0.001*		
[56-65]	0.94 [0.77-1.15]			
[66-74]	0.66 [0.54-0.81]			
[75-92]	0.78 [0.63-0.96]			
**GENDER**				
Female	1 (reference)	*<0.001*		
Male	0.47 [0.41-0.54]			
**TUMOUR SITE**				
Breast	1 (reference)	*<0.001*	1 (reference)	*<0.001*
Prostate	0.26 [0.22-0.32]		0.27 [0.22-0.34]	
Lung	0.78 [0.65-0.94]		0.77 [0.64-0.93]	
Sarcoma	0.46 [0.32-0.65]		0.46 [0.33-0.66]	
Colorectal	0.67 [0.55-0.82]		0.67 [0.55-0.82]	
**ECOG PS**				
0	1.2 (1.04-1.39)	*0.011*		
1	1 (reference)			
2	1.47 (1.13-1.91)			
>2	1.44 (0.92-2.25)			
**QUINTILE EDI**				
1	0.92 [0.75-1.14]	*0.02*	0.96 [0.78-1.19]	*0.429*
2	0.79 [0.65-0.96]		0.88 [0.74-1.06]	
3	0.79 [0.65-0.96]		0.86 [0.71-1.04]	
4	1.01 [0.84-1.22]		0.99 [0.83-1.20]	
5	1 (reference)		1 (reference)	
**T staging**				
1	1 (reference)	*0.001*		
2	0.83 [0.71-0.97]			
3	0.71 [0.59-0.86]			
4	0.72 [0.55-0.93]			
**N staging**				
0	1 (reference)	*0.037*		
1	1.23 [1.04-1.44]			
2	1.27 [1.02-1.58]			
3	1.03 [0.7-1.52]			
**M status**				
0	1 (reference)	*0.741*		
1	1.03 [0.87-1.21]			

Univariate associations between patients' characteristics and Time-To-Treatment are reported on Table 3. EDI was not associated with Time-To-Treatment (*p* = 0.324). After elimination of correlated variables, a multivariate analysis (Table 3) identified the tumor site as the only independently associated factor with Time-To-Treatment: patients with breast cancer have a shorter time to treatment than patients with prostate cancer (adjusted HR 0.32 [0.27-0.39], *p* < 0.001).

**Table 3:** Time-To-Treatment univariate and multivariate analyses

	Time to treatment			
	Univariate analysis		Multivariate analysis	
	HR [95%CI]	*p value*	AHR [95%CI]	*p* value
**AGE**				
[19-55]	1 (reference)	*<0.001*		
[56-65]	0.72 [0.6-0.87]			
[66-74]	0.6 [0.51-0.72]			
[75-92]	0.73 [0.61-0.88]			
**GENDER**				
Female	1 (reference)	*<0.001*		
Male	0.46 [0.4-0.53]			
**TUMOUR SITE**				
Breast	1 (reference)	*<0.001*	1 (reference)	*<0.001*
Prostate	0.32 [0.26-0.39]		0.32 [0.27-0.39]	
Lung	0.76 [0.63-0.93]		0.75 [0.62-0.92]	
Sarcoma	0.7 [0-5.1]		0.7 [0.49-0.99]	
Colorectal	0.81 [0.66-0.99]		0.81 [0.66-0.99]	
**ECOG PS**				
0	0.56 [0.36-0.86]	*0.046*		
1	0.53[0.34-0.83]			
2	0.65 [0.4-1.06]			
>2	1 (reference)			
**QUINTILE EDI**				
1	1 (reference)	*0.324*	1 (reference)	*0.982*
2	0.84 [0.67-1.05]		0.98 [0.78-1.22]	
3	0.98 [0.78-1.23]		1.04 [0.82-1.31]	
4	0.99 [0.79-1.24]		0.99 [0.79-1.2]	
5	1.0023 [0.81-1.24]		0.98 [0.79-1.21]	
**T staging**				
1	1 (reference)	*<0.001*		
2	0.81 [0.7-0.95]			
3	0.84 [0.7-1.02]			
4	1.54 [1.2-2]			
**N staging**				
0	1 (reference)	*<0.001*		
1	1.33 [1.1-1.6]			
2	1.5 [1.2-1.8]			
3	1.2 [0.8-1.8]			
**M status**				
0	1 (reference)	*<0.001*		
1	1.58 [1.34-1.87]			

Strata analyses (Table 4) did not manage to identify any association between EDI-quintile and Time-To-Diagnosis for any tumor site explored. T-staging at baseline was statistically associated with Time-To-Diagnosis for breast cancer, after a multivariate analysis (*p* < 0.001). Age at diagnosis and T-staging at baseline were statistically associated with Time-To-Diagnosis for prostate cancer, after multivariate analysis (*p* = 0.034 and *p* = 0.034, respectively). Metastases at baseline and performance status were associated with Time-To-Diagnosis for Sarcoma (*p* = 0.002 and *p* = 0.009 respectively). After adjustment, no variables were found independently associated to Time-To-Diagnosis for patients with lung cancer or colorectal cancer.

**Table 4:** strata analysis results : independents factors associated with Time to diagnosis, identified by multivariate analyses, for each tumor site

	Breast cancer				Prostate cancer				Sarcoma				Lung cancer				Colorectal cancer			
	univariate		multivariate		univariate		multivariate		univariate		multivariate		univariate		multivariate		univariate		multivariate	
	HR [95%CI]	*p* (LR)	HR [95%CI]	*p* (LR)	HR [95%CI]	*p* (LR)	HR [95%CI]	*p* (LR)	HR[CI]	*p* (LR)	AHR [CI]	*p* (LR)	HR [95%CI]	*p* (LR)	AHR [95%CI]	*p* (LR)	HR [95%CI]	*p* (LR)	AHR [95%CI]	*p* (LR)
**AGE**					0.68 [0.28-1.62]		0.71 [0.27-1.9]		1 (reference)	0.845			1 (reference)	0.726			1 (reference)	0.879		
[19-55]	1 (reference)	0.419			1 (reference)	0.04	1 (reference)	**0.034**	0.66 [0.23-1.9]				0.86 [0.52-1.43]				0.98 [0.54-1.77]			
[56-65]	1.17 [0.94-1.47]				0.54 [0.36-0.81]		0.52 [0.33-0.8]		1.11 [0.35-3.57]				1.05 [0.64-1.72]				1.17 [0.65-2.12]			
[66-74]	1.19 [50.92-1.53]				0.67 [0.43-1.04]		0.55 [0.34-0.9]		0.84 [0.37-1.89]				1.1 [0.62-1.94]				1.12 [0.62-2]			
[75-92]	1.12 [0.87-1.44]																			
**GENDER**																				
Female	1 (reference)	0.649			NA	NA			1 (reference)	0.172	1 (reference)	0.623	1 (reference)	0.848			1 (reference)	0.89		
Male	1.32 [0.42-4.11]								0.6 [0.29-1.23]		0.78 [0.29-2.08]		1.04 [0.73-1.47]				0.97 [0.67-1.42]			
**ECOG PS**																				
0	1 (reference)	0.287			1 (reference)	0.005			1 (reference)	0.09	1 (reference)	**0.009**	1 (reference)	0.102	1 (reference)	0.359	1 (reference)	0.433		
1	0.81 [0.64-1.02]				0.84 [0.59-1.2]				0.41 [0.18-0.92]		0.23 [0.09-0.61]		1.26 [0.79-2.01]		1.47 [0.8-3.38]		1.4 [0.92-2.14]			
2	1.14 [0.64-2.03]				2.59 [1.38-4.87]				1.29 [0.34-4.89]		1.01 [0.2-5.2]		1.76 [1.02-3.03]		1.75 [0.82-3.75]		1.32 [0.76-2.31]			
>2	0.79 0.33-1.92]				4.54 [1.41-14.66]				0.25 [0.03-1.99]		0.1 [0.01-1.71]		2.27 [1.01-5.1]		2.76 [0.71-10.66]		1.17 [0.42-3.24]			
**QUINTILE EDI**																				
1	1 (reference)	0.628	1 (reference)	0.611	1 (reference)	0.103	1 (reference)	0.081	1 (reference)	0.68	1 (reference)	0.314	1 (reference)	0.994	1 (reference)	0.982	1 (reference)	0.755	1 (reference)	0.886
2	0.83 [0.61-1.14]		0.85 [0.62-1.16]		1.13 [0.67-1.92]		1.42 [0.8-2.51]		1.04 [0.26-4.24]		0.92 [0.12-7.28]		1.06 [0.59-1.89]		1.03 [0.46-2.32]		0.91 [0.47-1.77]		0.87 [0.42-1.78]	
3	0.9 [0.66-1.23]		0.97 [0.71-1.32]		0.85 [0.47-1.54]		1.03 [0.54-1.97]		0.74 [0.2-2.8]		0.85 [0.12-6.12]		0.95 [0.52-1.75]		0.97 [0.44-2.13]		0.88 [0.44-1.75]		0.9 [0.43-1.9]	
4	0.97 [0.72-1.31]		1.01 [0.75-1.37]		1.72 [0.96-3.06]		1.91 [1.02-3.58]		1.8 [0.41-7.78]		3.56 [0.49-25.9]		1.0 [0.56-1.81]		1.2 [0.54-2.67]		0.69 [0.34-1.38]		0.73 [0.34-1.53]	
5	1.01 [0.77-1.34]		1.05 [0.79-1.4]		1.29 [0.74-2.25]		1.79 [0.93-3.43]		0.85 [0.23-3.11]		1.11 [0.14-8.8]		0.95 [0.55-1.65]		1.13 [0.53-2.38]		0.98 [0.53-1.81]		0.97 [0.5-1.9]	
**T staging**																				
1	1 (reference)	<0.001	1 (reference)	**<0.001**	1 (reference)	0.153	1 (reference)	**0.034**	1 (reference)	0.174			1 (reference)	0.171	1 (reference)	0.145	1 (reference)	0.222		
2	0.8 [0.66-0.98]		0.79 [0.64-0.97]		1.51 [1.05-2.17]		1.61 [1.07-2.45]		NA				1.7 [0.92-3.14]		1.7 [0.86-3.38]		0.38 [0.09-1.54]			
3	0.53 [0.33-0.83]		0.52 [0.33-0.81]		1.29 [0.83-2.01]		1.42 [0.86-2.34]		NA				1.26 [0.67-2.4]		1.24 [0.61-2.53]		0.32 [0.1-1.05]			
4	0.39 [0.23-0.64]		0.36 [0.21-0.61]		2.37 [0.32-17.37]		2.2 [0.3-16.69]		NA				0.96 [0.49-1.91]		0.83 [0.39-1.76]		0.25 [0.08-0.85]			
**N staging**		0.021																		
0	1 (reference)				1 (reference)	0.038			1 (reference)	0.414			1 (reference)	0.707			1 (reference)	0.141	1 (reference)	0.189
1	0.87 [0.71-1.06]				2.54 [1.16-5.55]				0.91 [0.16-5.1]				1.16 [0.57-2.35]				0.66 [0.42-1.06]		0.67 [0.42-1.09]	
2	0.8 [0.56-1.16]				NA				7.31 [0.44-121]				1.37 [0.78-2.41]				0.97 [0.55-1.73]		0.95 [0.52-1.76]	
3	0.43 [0.22-0.84]				NA				NA				1.31 [0.67-2.57]				NA		NA	
**M status**		0.031				<0.001														
0	1 (reference)				1 (reference)				1 (reference)	0.125	1 (reference)	**0.002**	1 (reference)	0.62			1 (reference)	0.828		
1	0.62 [0.39-0.99]				3.29 [1.89-5.72]				0.6 [0.29-1.23]		0.21 [0.07-0.62]		0.92 [0.65-1.29]				1.04 [0.71-1.52]			

Strata analyses (Table 5) did not manage either to identify association between deprivation and Time-To-Treatment, for any of the tumor sites explored. For prostate cancer, the health condition estimated by performance status was independently associated with a short Time-To-Treatment if it was good (PS = 1) (*p* < 0.001). Similarly, the smaller the T staging was, the shorter was the Time-To-Treatment (*p* = 0.045). For colorectal cancer, few factors were identified as influencing the Time-To-Treatment: Being a man lead to a quicker treatment response (*p* < 0.001), as well as having a small T staging (*p* = 0.006). Neither for sarcoma nor for breast cancer or lung cancer, were any factors identified as influencing Time-To-Treatment.

**Table 5:** strata analysis results : independents factors associated with Time to Treatment identified by multivariate analyses, for each tumor site

	Breast cancer				Prostate cancer				Sarcoma				Lung cancer				Colorectal cancer			
	univariate		multivariate		univariate		multivariate		univariate		multivariate		univariate		multivariate		univariate		multivariate	
	HR [95%CI]	p [LR]	HR [95%CI]	p [LR]	HR [95%CI]	p [LR]	HR [95%CI]	p [LR]	HR[CI]	p [LR]	AHR [CI]	p [LR]	HR [95%CI]	p [LR]	AHR [95%CI]	p [LR]	HR [95%CI]	p [LR]	AHR [95%CI]	p [LR]
**AGE**					1 [reference]	0.21			1 [reference]	**0.08**			1 [reference]	0.87			1 [reference]	0.63		
[19-55]	1 [reference]	0.66			0.82 [0.34-1.95]				1.01 [0.35-2.89]				0.92 [0.56-1.51]				0.94 [0.52-1.71]			
[56-65]	0.87 [0.69-1.09]				0.84 [0.37-1.94				0.29 [0.09-0.92]				0.9 [0.56-1.45]				1.14 [0.63-2.07]			
[66-74]	0.90 [0.7-1.15]				1.22 [0.52-2.87]				0.46 [0.19-1.08]				0.79 [0.46-1.37]				0.82 [0.45-1.49]			
[75-92]	0.94 [0.74-1.21]																			
**GENDER**																				
Female	1 [reference]	0.45			NA	NA			1 [reference]	0.94			1 [reference]	0.31			1 [reference]	**0.003**	1 [reference]	**<0.001**
Male	0.67 [0.21-2.08]								0.97 [0.47-1.99]				0.83 [0.58-1.19]				0.54 [0.36-0.81]		0.45 [0.29-0.71]	
**ECOG PS**																				
0	1.15 [0.47-2.82]	0.66			0.06 [0.02-0.2]	**<0.001**	0.04 [0.01-0.2]	**<0.001**	0.91 [0.11-7.29]	0.64			0.57 [0.26-1.25]	0.38			0.96 [0.35-2.68]	0.52		
1	0.90 [0.39-2.48]				0.06 [0.02-0.21]		0.04 [0.01-0.17]		0.61 [0.08-4.74]				0.62 [0.3-1.29]				1.02 [0.36-2.9]			
2	1.12 [0.39-3.24]				0.27 [0.07-1.01]		0.21 [0.04-1.17]		0.44 [0.04-4.41]				0.49 [0.22-1.08]				1.53 [0.5-4.67]			
>2	1 [reference]				1 [reference]		1 [reference]		1 [reference]				1 [reference]				1 [reference]			
**QUINTILE EDI**																				
1	1 [reference]	0.49			1 [reference]	0.87	1 [reference]	0.58	1 [reference]	0.67			1 [reference]	0.74	1 [reference]	0.63	1 [reference]	0.32	1 [reference]	0.93
2	0.83 [0.62-1.09]				0.9 [0.53-1.52]		0.84 [0.48-1.47]		0.72 [0.18-2.95]				0.74 [0.41-1.34]		0.74 [0.41-1.34]		1.42 [0.73-2.78]		1.29 [0.63-2.66]	
3	1.02 [0.75-1.38]				1.05 [0.58-1.88]		1.01 [0.55-1.88]		0.6 [0.16-2.32]				0.75 [0.41-1.39]		0.73 [0.39-1.35]		2.13 [1.06-4.31]		1.32 [0.63-2.77]	
4	0.97 [0.71-1.35]				0.86 [0.47-1.55]		0.73 [0.38-1.41]		1.51 [0.35-6.42]				0.8 [0.44-1.44]		0.76 [0.42-1.39]		1.61 [0.8-3.23]		1.08 [0.51-2.27]	
5	0.89 [0.66-1.21]				1.07 [0.62-1.85]		1.11 [0.61-2.01]		0.83 [0.22-3.07]				0.96 [0.55-1.66]		0.99 [0.57-1.72]		1.5 [0.8-2.79]		1.17 [0.6-2.29]	
**T staging**																				
1	1 [reference]	0.47			1 [reference]	**0.042**	1 [reference]	**0.045**	NA	NA			1 [reference]	0.13			1 [reference]	**0.017**	1 [reference]	**0.006**
2	1.0 [0.82-1.77]				1.21 [0.84-1.74]		1.34 [0.92-1.95]		NA				1.28 [0.69-2.37]				0.84 [0.21-3.41]		0.23 [0.07-0.82]	
3	0.88 [0.56-1.38]				1.83 [1.17-2.85]		2 [1.25-3.21]		NA				0.68 [0.36-1.31]				1.6 [0.5-5.14]		0.3 [0.12-0.76]	
4	1.48 [0.92-2.46]				5.87 [0.79-43.74]		1.15 [0.13-10.33]		NA				1.03 [0.52-2.04]				2.68 [0.81-8.85]		0.54 [0.34-0.85]	
**N staging**		0.3																		
0	1 [reference]				1 [reference]	**<0.001**			1 [reference]	0.7			1 [reference]	0.6			1 [reference]	0.12		
1	0.93 [0.76-1.15]				5.14 [2.34-11.32]				0.52 [0.1-2.81]				1.26 [0.62-2.57]				0.8 [0.5-1.26]			
2	1.22 [0.85-1.77]				NA				0.55 [0.06-5.05]				0.98 [0.57-1.71]				1.39 [0.78-2.47]			
3	1.65 [0.85-3.21]				NA				NA				0.77 [0.4-1.49]				NA			
**M status**		0.34				**<0.001**														
0	1 [reference]				1 [reference]				1 [reference]	0.29			1 [reference]	**0.09**	1 [reference]	**0.07**	1 [reference]	**0.003**		
1	1.26 [0.8-2.0]				7.39 [4.11-14.02]				1.59 [0.7-3.64]				1.34 [0.95-1.9]		1.38 [0.97-1.96]		1.82 [1.23-2.69]			

## DISCUSSION

In this exhaustive, mono-centric population-based study, the primitive tumor site seems the main predictive factor for Time-to-diagnostic and Time-To-Treatment. The Socio Economic Status, estimated with the European District Index, has not been identified as modifying Time-To-Diagnosis or Time-To-Treatment in this population. Even for inside strata analyses, that removed the strong tumor site effect, deprivation does not appear as a factor influencing Time-To-Diagnosis and Time-To-Treatment.

According to the tumor site, times could vary in the ratio of one to 6 for Time-To-Diagnosis : 0.75 month for breast cancer and 4.69 months for prostate cancer and of one to 3 for Time-To-Treatment : 0.75 month for breast cancer and 2.02 months for prostate cancer. These different observed times illustrate the heterogeneity of evolution and practices according to the tumor site:

Time-To-Diagnosis is shorter for breast cancer because of the screening practice, which shortens symptoms onset and which is the first step into a relatively standardized, prompt care path extending from first suspicion to completion of all treatments. On the contrary, the prostate cancer care path requires more time, since elevated prostatic specific antigen (PSA) generally lead first to a watchful follow-up rather than to an immediate biopsy, within the context of a slow growing tumor.

Time-To-Treatment has been established as a quality of care indicator worldwide : guidelines have been produced to regulate its maximum limit [27, 28]. It has been estimated in this study from 0.34 month for the sarcomas (featuring an emergency response to cancer) to 2.02 months for the prostate cancer, which is considered as an indolent cancer. The median Time-To-Treatment is 0,9 months (27,2 days) for all cancers in this study, which is under the threshold established by guidelines reporting the preferable delays for treatment [27, 28]: 28 days in the USA and 31 days in the UK. If prostate cancer is excluded, all others tumor sites present an adequate speed of treatment response (from 0.34 to 0.79 month).

Tumor site strata analyses brought some information: The medical factors related to the disease (T staging for breast cancer and presence of metastases for sarcoma) confirmed what is observed in practice : the more symptomatic the tumor is, the sooner it is detected. Other factors (performance status, metastatic status) appear to have been taken into account concerning the decision to treat the process so that the best and least harmful care path can be scheduled. We found no explanation for the difference in Time-To-Treatment between woman and men for colorectal cancer. This needs to be further explored. What's more, a lack of power into these strata analyses must be considered and does not allow us to conclude firmly.

This study is the first to explore elapsed times from symptoms to first treatment in 5 cancers, and in France. Its strength resides in the setting of the data collection: The sample was an absolute exhaustive collection of patients: All patients admitted to the Cancer Comprehensive Center, for one year, were suffering from the 4 more frequent and one rare cancers, with no missing data. What's more, this sample can be considered as highly representative of a rural French population because of the particular recruitment of this center: Being the only center in this wide area with both oncologic and radio-therapeutic departments, this center is in monopoly in the Loire County. No selection bias can be here reported. This study is also one of the first to use the new French EDI to assess Socio-economic deprivation.

This study didn't identify any association between Time-To-Diagnosis / Time-To-Treatment and socio-economic deprivation. In a review of studies examining the association of cancer survival with socio-economic status, Woods *et* al [32] identified 38 articles and mentioned 14 studies having reported no association between socio-economic status and cancer survival. What's more, in ecological studies using deprivation index, differences were systematically smaller, due to the inaccuracy of the estimate. Even in studies identifying an impact of the socio-economic status on survival, the estimated effect was often moderate. More recent studies confirmed this tendency : individual deprivation estimates [33] lead to a stronger association than ecological ones [34, 35]. All of the recent studies without any association between socio-economic deprivation and cancer outcomes used the ecological Index, either to explore relationships with cancer survival [36], cancer treatment [8, 37], or HPV vaccination [38].

These results lead to this assumption: an ecological index is not such a good estimate for the individual patient socioeconomic status. The deprivation score for an area cannot apply to all its residents. People can live in a deprived area, without being themselves deprived. What's more the French EDI is a new index, which has not demonstrated its psychometric validity on the long term for now. This Index has been used in this French area (Loire county) which is mostly a rural county. Yet, Bertin et al, demonstrated recently that a deprivation index was less valid in rural places [39]: the homogeneity for socioeconomic status in each geographical area is less existent.

Another assumption can be that the cancer outcomes chosen were not relevant. Yet these outcomes have already been identified as quality-of-care indicators as well as proxy for evolution and survival [27, 28, 40, 41]. Saint Jacques et al and Dalton et al succeeded to identify an association between longer Time-To-Treatment and low educational level for breast cancer [42], and lung cancer [25]. Berglund underlined an impact of the socioeconomic status (measured by combining income, education level and occupation) on the Time-To-Treatment for lung cancer [43]. So these indicators seemed theoretically relevant as cancer end points to explore.

The last assumption could be that in this particular setting, the socioeconomic status did not impact the waiting times. But we believe the explanation should be searched for elsewhere, in an index that measures socioeconomic deprivation more appropriately, probably with individual level data.

In practice, this study underlined the difficulty to assess individual socio-economic level, and by that, to assess its impact on cancer-related health outcomes. The only postal address seams not sufficient, at least for such a rural area, to provide an exact overview of inequalities. If health care inequalities are considered by the competent authorities to be mandatory issues to be tackle, the hospital routine data collection should require additional characteristics to be gathered, for each patient. Those characteristics could allow a global evaluation of the patient beyond its medical condition. What those extra data should be composed of remains to be settle down.

Our study faced several limitations: More variables could have been studied, but we did not access more, in an exhaustive way. But even with these few, we identified other factors influencing the Time-To-Diagnosis and the Time-To-Treatment. We can assume that with even more variables, we would have had the same results. Our study is a monocentric study, which shortens the specter of representation. Yet as we said above, the patients admitted to this center are particularly diverse and representative of the whole county population. As mentioned previously, an ecological geocoded index (EDI) has been used to estimate patients' deprivation. This geographical estimation may not be enough relevant, either because the patients' sample is peculiar (people who live in rural areas), or because the EDI is a non-individual estimation. Labbe et al [44] recently demonstrated how a specific and individual French score (EPICES score) may be more reliable to diagnose deprivation. However, this score requires access to individual data that are usually unavailable in databases, while place of residence (allowing the use of the EDI) is systematically collected. Labbe et al proposed to aggregate this score to perform an ecological index: The ecological EPICES score could then be used to estimate, perhaps more precisely, French patients' deprivation. However, this score would not address the problem of comparison across countries which is allowed by the EDI. Thus, although less accurate, EDI may provide an opportunity in both assessing deprivation and comparison across settings. Five tumor sites have been explored in this study. It allows the results to give a fair representation of the medical practices of a cancer comprehensive center. Yet, this heterogeneous population led to a lack of power for all subgroup analysis.

Despite all these limits, the methodology used remains adequate and allows us to conclude on an absence of a major influence of the patients' deprivation estimated with the EDI on waiting times, but on a major influence of the tumor site on those waiting times, in this setting.

## CONCLUSIONS

Deprivation estimated by European deprivation Index French version, does not appear to be related to an extension of the time to diagnosis or time to treatment in our real-life population of cancer patients. Cancer location and the tumor staging at baseline are much more powerful factors explaining variation in waiting times. Waiting times estimated in this study are concordant with the maximum delay recommended for the cancer care worldwide, and translate good quality-of-care in this setting. Yet we cannot stop at these rather soothing results. Further research should be done to clearly identify and measure, at an individual-level, more sensitive frailty factors that could be responsible for delays in care. Developing actions targeting those fragile populations would be the next step.

## PATIENTS AND METHODS

### Design

A retrospective mono-centered cohort study was performed.

### Population

We included retrospectively all consecutive incoming adult patients diagnosed for breast cancer (BC), prostate cancer (PC), colorectal cancer (CRC), lung cancer (LC) or sarcoma (S) and admitted to the comprehensive cancer centre of Saint Etienne (France) between the 1st of January 2013 and the 31st of December 2013. BCs were both ductal carcinoma and adenoma carcinoma. CRCs were defined as cancer arising from the cecum to the rectum. LC includes small cell carcinoma and non-small cell carcinoma. S included soft tissue sarcoma, osteosarcoma and Darrier-Ferrand syndrome. Patients were included only if they had a confirmed histological malignancy. Patients were excluded if they had tumor relapse, hematological malignancies or other tumor site.

### Data collection

Once included, data for all patients were collected from two databases: demographical data were collected from the administrative database and Medical data were collected from the medical information system of the Institute. Missing data were gathered from individual medical files.

Clinical data were gathered : clinical condition status at baseline using Eastern Cooperative Oncology Group (ECOG) performance status (PS), cancer extension according to the TNM staging and grading (Scarf Bloom and Richardson, SBR grade for patients with BC, Gleason score for patients with PC, grading for patients with sarcoma). Symptomatology history was reconstructed by the clinician with direct examination during the first consultation. Onset of symptoms was collected according to the type of cancer : date of first clinical signs (pain, tumor hardening, dysuria, hematuria, cough, hemoptysis, rectal bleeding…), or date of the first abnormal radiological or biological result for cancer screening (abnormal mammography, colonoscopy, PSA increase). Consecutive treatments were reported, as well as follow up. Patient socio-demographic characteristics and postal address were also recorded.

Date of diagnosis was defined as the date of the first histological proof of malignancies (by biopsy or cytological, otherwise surgical sample). Time-To-Diagnosis was the time between onset of symptoms and date of diagnosis. Time-to-treatment was the time between the date of diagnosis and the date of the first specific treatment (surgery, chemotherapy, hormone therapy, radiation therapy, surveillance or best supportive cares).

### French EDI

The EDI is an adaptable transnational ecological deprivation index. It has been developed according to a common definition of deprivation- physical and social- while maintaining the specificity of each country. This index combines on one hand, individual data from a European survey on poverty launched by the European Commission (EU-SILC) [31], and in the other hand, data from the population census of each country. Those characteristics (identical study design for all countries, dynamic cohort), allows this index to be transposable in time and from one country to another and should help in characterize and compare socio-economic characteristics of a population across settings. This ecological deprivation index has been built and used for the first time in France in 2012 [31, 32]. The EDI can be replicated in 24 other European countries. The French EDI is divided in 5 quintiles; from 1 (least deprived) to 5 (most deprived).

The deprivation level for each patient was estimated by assigning him the EDI quintile of his place of house, deducted from his postal address (the only geographical data we could retrospectively gathered). Deprivation was therefore estimated rather than individually assessed. The postal address of each patient was geocoded on global positioning system (GPS) coordinates using Google Map^®^ (Google Inc, California, USA); these coordinates were linked to an EDI quintile using an area-based measure, which was attributed to the patient.

### Statistical analysis

Descriptive statistics were used to characterize patients' population (median and inter-quartile range for continuous variables; frequencies and proportions for categorical variables; percentage of missing data). Time-To-Diagnosis and Time-To-Treatment were estimated using a censored data model. The unadjusted associations between patients' characteristics and Time-To-Diagnosis as well as Time-To-Treatment were tested with log-rank comparisons. Multivariate analyses were performed using Cox regression. Variables with a *p* < 0.20 in the univariate analyses were included in the multivariate analyses with a significance threshold of *p* < 0.05. The association of EDI and Time-To-Diagnosis, as well as Time-To-Treatment was analyzed by tumor site strata. Statistical analyses were performed with R 3.0.2
